# Assessment of diaphragmatic function parameters by intensive care ultrasound compared to conventional parameters during spontaneous breathing trial

**DOI:** 10.1186/2197-425X-3-S1-A1004

**Published:** 2015-10-01

**Authors:** P Theerawit, D Eksombatchai, Y Sutherasan, T Suwatanapongched, S Kiatboonsri

**Affiliations:** Ramathibodi Hospital, Bangkok, Thailand

## Introduction

Ultrasound can demonstrate the characteristics of the diaphragm functions that involve to the weaning process.

## Objectives

Our primary objective is to study the performance of the diaphragmatic function parameters assessed by ultrasound regarding the weaning outcomes.

## Methods

This is a prospective observational study conducted between June 2013 and November 2013 in intensive care unit patients planned to wean with spontaneous breathing trial. After 1-hour of T-piece trial, the patient's RSBI, TV, VC and NIP were recorded. By ultrasound, we measured the amplitude of diaphragmatic movement (ADM), time to the maximum amplitude (TTMA), and the diaphragmatic thickness (DT).

## Results

A total of 62 patients were enrolled. The ADM was correlated with RSBI and TV (*r* = -0.435 and *r* = 0.445; *P* < 0.001). The TTMA was correlated with RSBI, TV and VC (*r* = -0.703, *r* = 0.639 and *r* = 0.522; *P* < 0.001). The Right delta thickness was correlated with RSBI (*r* = -0.26; *P* = 0.04).The TTMA predicted weaning failure with an area under the receiver operator characteristic curve (ROC) by 0.698 comparable to ROC of RSBI (0.659). The amplitude and the delta thickness of right diaphragm predicted re-intubation with the ROC by 0.723 and 0.817 respectively.

## Conclusions

Diaphragmatic function parameters assessed during spontaneous breathing trial was significantly correlated with conventional weaning parameters. The performance of the TTMA was comparable to that of the RSBI for predicting weaning failure. Additionally the right diaphragmatic delta thickness can predict the re-intubation within 48 hours.

## References

[1] Kim WY, Suh HJ, Hong SB, Koh Y, Lim CM (2011). Diaphragm dysfunction assessed by ultrasonography: influence on weaning from mechanical ventilation. Crit Care Med.

[2] Matamis D, Soilemezi E, Tsagourias M, Akoumianaki E, Dimassi S, Boroli F, Richard JC, Brochard L (2013). Sonographic evaluation of the diaphragm in critically ill patients. Technique and clinical applications. Intensive Care Med.

